# Single-cell analysis identifies a VIM+ glioma stem-like vascular–immune state linked to myeloid OSM signaling and CEBPD activity

**DOI:** 10.3389/fimmu.2026.1863737

**Published:** 2026-08-11

**Authors:** Qingbao Xiao, Bo Chen, Junhao Yan, Cheng Li, Jian Xu, Mingrui Luo, Bin Wang, Yuan Zhu

**Affiliations:** 1 Department of Neurosurgery, Wuhan Third Hospital, Tongren Hospital of Wuhan University, Wuhan, Hubei, China; 2 The Third Clinical College, Henan Medical University, Xinxiang, China; 3 Department of Neurosurgery, First Affiliated Hospital of Anhui Medical University, Hefei, China

**Keywords:** CEBPD, glioma, glioma stem-like tumor cells, IDH-wild-type, vascular–immune crosstalk

## Abstract

**Background:**

Gliomas show intertumoral and intratumoral heterogeneity, and isocitrate dehydrogenase (IDH) status is linked to biological behavior and clinical aggressiveness. Evidence suggests glioma progression depends on reciprocal crosstalk between glioma stem-like tumor cells (GSTCs) and the tumor microenvironment, where immune and vascular remodeling promote plasticity, invasion, and immune evasion. However, GSTC state architecture across IDH backgrounds and its links to vascular–immune cues remain unclear. We aimed to define IDH-associated GSTC states and their functional and regulatory features.

**Methods:**

Single-cell RNA-sequencing data from 33 glioma patients (GSE278456) were analyzed using Seurat integration, clustering, differential expression analysis, and inferCNV-assisted malignant cell identification. Reclustered GSTCs were assessed by Gene Ontology (GO)/Hallmark enrichment, Area Under the Curve-based Cell-wise Gene Set Enrichment Analysis (AUCell), CytoTRACE, Monocle, Slingshot, CellChat, and pySCENIC to characterize states, developmental trajectories, cell-cell communication, and regulatory networks. After CEBPD silencing in U251 and LN229 cells, quantitative real-time PCR (qRT-PCR), Cell Counting Kit-8 (CCK-8), colony formation, Transwell migration, and apoptosis assays were performed.

**Results:**

Six cell populations and five GSTC subgroups were identified. A VIM+ GSTC state (C0), enriched in IDH-wild-type tumors, highly expressed CHI3L1, VIM, LGALS3, VEGFA, and SEC61G. Compared with other states, C0 showed stronger angiogenesis, hypoxia, IL6/JAK/STAT3 signaling, and epithelial–mesenchymal transition-related activities, with enrichment of oxidative phosphorylation, focal adhesion, cell-substrate junction, and actin/cadherin-binding programs. Trajectory analyses placed C0 at an early-to-intermediate node linked to divergent later states, indicating plasticity distinct from a purely proliferative state. CellChat suggested prominent myeloid-to-C0 communication, with OSM as a candidate input and LIFR/IL6ST as the receptor basis. pySCENIC identified a C0-preferential regulon landscape centered on CEBPD, RUNX2, POU2F3, TEAD3, and TEAD4, plus an M2 regulon module associated with stress adaptation and intracellular homeostasis. CEBPD knockdown reduced proliferation, colony formation, and migration while increasing apoptosis.

**Conclusion:**

We identify an IDH-wild-type-enriched VIM+ glioma stem-like state with angiogenic, hypoxic, mesenchymal-like, and inflammatory features, and nominate myeloid-derived OSM signaling and CEBPD as candidate mediators of this vascular–immune-linked malignant phenotype. These findings provide a hypothesis-generating, state-based perspective on glioma progression across IDH backgrounds and highlight candidate biomarkers and mechanistic entry points for validation and therapeutic stratification.

## 1 Introduction

Gliomas, particularly high-grade gliomas and glioblastoma (GBM), are among the most aggressive primary malignant tumors of the central nervous system in adults (1). Current standard treatment remains based on surgical resection, radiotherapy, and temozolomide chemotherapy, but its overall therapeutic efficacy is limited (1). Treatment remains challenging not only because of the extensive infiltration of tumors into the surrounding brain tissue and the restrictions imposed by the blood-brain barrier on systemic therapy, but also because of the pronounced heterogeneity and high recurrence rate of these tumors (1, 2). Increasing evidence indicates that intratumoral heterogeneity remains a critical obstacle to effective treatment and a major driver of tumor recurrence and therapeutic resistance. At the same time, treatment strategies for brain tumors are themselves highly dependent on factors such as tumor type, location, grade, and the status of surrounding tissues. With advances in genomic sequencing and molecular diagnostics, more refined stratification based on molecular features is gradually becoming an important foundation for optimizing individualized treatment strategies (3, 4). Noninvasive classification based on imaging features is also continuously evolving. The introduction of quantitative susceptibility mapping (QSM) combined with deep learning on the basis of conventional magnetic resonance imaging (MRI) has improved the performance of glioma grading as well as the discrimination of IDH1 mutation and ATRX loss compared with conventional MRI alone (5). In GBM, this heterogeneity exists not only within tumor cells themselves but is also reflected in an immunosuppressive microenvironment characterized by myeloid-cell enrichment and limited lymphocyte infiltration, as well as in the sustained and complex interactions between tumor cells and surrounding microenvironmental components, including neurons and glial cells (6–8). Meanwhile, GBM is often accompanied by marked vascular hyperplasia, suggesting that changes in malignant cell states and microenvironmental remodeling are intertwined and jointly contribute to tumor progression (6–8).

In the molecular stratification of gliomas, IDH status is closely associated with tumor biological behavior. Previous studies have shown that the aggressiveness of high-grade gliomas is largely driven by marked heterogeneity, and that in IDH-wild-type (IDH-WT) tumors, increased intratumoral heterogeneity is associated with a more aggressive disease phenotype (9). At the same time, current understanding of GBM indicates that its progression involves the combined effects of multiple factors, including genetic alterations, epigenetic regulation, hypoxic adaptation, metabolic reprogramming, immune evasion, and glioma stem cell-related states (10). From a broader developmental signaling perspective, the Wnt pathway is involved in stem cell self-renewal, differentiation, and epithelial–mesenchymal transition (EMT)-related processes, further suggesting that the establishment of malignant glioma states is more likely to result from the reorganization of multilayered state programs rather than from a simple increase in proliferation alone (11). Studies focusing on the blood-brain barrier and the tumor vascular system have further shown that, compared with low-grade gliomas, GBM exhibits more pronounced pathological vascular cell alterations, implying that distinct malignant states may be closely linked to microenvironmental remodeling (12). However, the internal state composition of glioma stem-like tumor cells (GSTCs), their positions along state transitions, and their key communication relationships with the myeloid microenvironment across different IDH backgrounds remain to be systematically elucidated.

To address this gap, the present study performed a discovery-oriented analysis of publicly available single-cell transcriptomic data to delineate the cellular composition of gliomas and the internal state heterogeneity of GSTCs across different IDH molecular backgrounds, with particular attention to a GSTC state relatively enriched in IDH-WT tumors. By integrating functional profiling, developmental trajectory inference, cell-cell communication analysis, and transcriptional regulatory network analysis, we characterized the mesenchymal-like, hypoxia-adaptive, inflammatory, and angiogenesis-related programs associated with this state, and further evaluated its potential linkage to myeloid-derived signals in a vascular–immune microenvironmental setting. Finally, through complementary *in vitro* experiments, we performed a target-level functional assessment of CEBPD, a candidate regulator nominated by the single-cell analyses, with the aim of providing a state-based framework for understanding malignant glioma progression across different IDH backgrounds and highlighting potential regulatory nodes relevant to microenvironment-associated malignant progression.

## 2 Methods

### 2.1 Data collection and processing

The single-cell RNA-sequencing (scRNA-seq) data analyzed in this study were obtained from the Gene Expression Omnibus (GEO; https://www.ncbi.nlm.nih.gov/geo/) under accession number GSE278456. Information on the samples included in the analysis is provided in **Supplementary Table 1**. In the R environment (v4.3.3), the Seurat package (v4.3.0) was used to read and process the single-cell RNA sequencing data from each sample. First, the DoubletFinder program (v2.0.3) was used to identify and remove potential doublets and low-quality cells. Cells meeting the following criteria were retained for subsequent analysis: 500 < nFeature_RNA < 6000 and a mitochondrial gene expression proportion of less than 25%. This mitochondrial threshold was selected considering the surgical glioma origin of the dataset and the possibility that overly stringent filtering could remove metabolically active malignant cells exposed to hypoxia, oxidative stress, tissue dissociation-related stress, or vascular remodeling. To reduce the retention of low-quality cells, mitochondrial filtering was not used as a stand-alone criterion, but was combined with nFeature_RNA restriction, DoubletFinder-based doublet removal, inspection of nFeature_RNA and nCount_RNA distributions, marker-based cell annotation, and inferCNV (Inference of Copy Number Variations). Following quality-control filtering, gene expression matrices from individual samples were normalized using the NormalizeData() function with the LogNormalize method and a scale factor of 10,000. The top 2,000 highly variable genes (HVGs) were identified using the FindVariableFeatures() function with the “vst” selection method. After data scaling, principal component analysis (PCA) was performed using the selected HVGs. To correct for batch effects across samples, the Harmony algorithm was applied to the PCA embeddings. The first 30 Harmony-corrected dimensions were subsequently used to construct a shared nearest neighbor (SNN) graph using the FindNeighbors() function. Cell clusters were identified using the FindClusters() function at a resolution of 0.5. Uniform Manifold Approximation and Projection (UMAP) was performed using the same 30 Harmony-corrected dimensions for visualization. The overall analytical framework was consistent with established single-cell transcriptomic workflows (13).

### 2.2 Visualization of differentially expressed genes and AUCell-based enrichment analysis

The FindAllMarkers function in Seurat was used under default parameters to identify differentially expressed genes (DEGs) in each cell population and cell subgroup based on the Wilcoxon rank-sum test, with a screening threshold of logFC > 0.25. Subsequently, the clusterProfiler (v4.6.2) and SCP (v0.4.8) packages were used to perform enrichment analysis of DEGs in order to evaluate their potential functions in different cell populations and cell subgroups. The enriched pathways for each cell population and cell subgroup were all obtained based on GO analysis. In addition, AUCell was used to evaluate the activity of stemness gene set and transcription factors in the scRNA-seq data in order to characterize the functional features of different cell states. For stemness genes, genes associated with stem cell characteristics were collected by integrating published studies with publicly available stem cell resources related to the diseases and species under study. Before scoring, genes that were not detected in our scRNA-seq dataset were removed, and duplicate genes were excluded. Finally, gene sets were generated for calculating stem cell characteristics. The final gene sets used for scoring included: ABCG2, BMI1, CD34, CD44, CTNNB1, EPAS1, EZH2, HIF1A, KDM5B, KLF4, LGR5, MYC, NANOG, NES, NOTCH1, POU5F1, PROM1, SOX2, TWIST1, ZFP42 and ZSCAN4.

### 2.3 Assessment of CNV levels

The inferCNV algorithm (v1.17.0) was used to assess CNV levels in cells. During tumorigenesis, the copy number karyotypic features of aneuploid cells can be used to distinguish non-tumor cells from malignant tumor cells. Therefore, in this study, endothelial cells were used as the CNV reference population to determine whether other tumor cells exhibited significant chromosomal copy number variations.

### 2.4 Construction of pseudotime trajectories

The Monocle package (v2.24.0) was used to construct pseudotime trajectories based on the scRNA-seq expression profiles of GSTCs in order to reconstruct their continuous state transition process and identify the relative positions of different cell subgroups along the trajectory. Considering that Monocle is more suitable for depicting pseudotime ordering and state transition positions during continuous state changes, rather than independently defining the undifferentiated degree of cells, this study combined it with CytoTRACE and Slingshot for cross-evaluation of the developmental positions of GSTCs. The analytical strategy of combining multiple trajectory inference tools to help define the relative stage positions and branch relationships of cells has been applied in other single-cell studies of tumors (14–17).

### 2.5 CytoTRACE and Slingshot analysis

To investigate differences in developmental and differentiation states among different GSTC subgroups in glioma, CytoTRACE was used to rank the relative degree of differentiation of each tumor cell subgroup, and Slingshot was further used to infer their potential branched evolutionary trajectories as well as the dynamic changes of relevant differentially expressed genes along trajectory progression. Similar analytical strategies have been used in other tumor single-cell studies, in which specific cell lineages are re-clustered and then further combined with AUCell, pseudotime analysis, and cell-cell communication inference to characterize state heterogeneity and its relationship with the microenvironment. Accordingly, in this study, the developmental positions of GSTCs and their external signal inputs were jointly evaluated (18, 19).

### 2.6 Cell-cell communication

Based on the scRNA-seq data, the CellChat package (v1.6.1) was used to infer potential ligand–receptor communication among different cell types, including communication between tumor cell subgroups and microenvironmental cells. CellChatDB.human was used as the reference database for ligand–receptor interactions, and a P value cutoff of 0.05 was applied as the significance threshold. In this study, CellChat-derived events were interpreted as computationally inferred candidate communication axes based on ligand–receptor co-expression patterns, rather than as direct evidence of functional interaction, receptor activation, or spatial proximity between the predicted sender and receiver populations.

In this study, we performed more detailed visualization of the interactions between key tumor cell subgroups and relevant immune/stromal cells to help identify potential key signaling pathways. At the same time, the tumor immune microenvironment is not composed solely of immune cells, but also involves cytokines, extracellular matrix (ECM) components, and vesicle-mediated molecular exchange; in other solid tumors, myeloid-derived suppressor cells (MDSCs), regulatory T cells (Tregs), tumor-associated macrophages (TAMs), and inhibitory soluble factors are also commonly included in the framework of the immunosuppressive niche. Therefore, including both immune cells and stromal-related components in communication analysis helps provide a more complete assessment of the external inputs to GSTCs (20, 21).

Considering that, in non-tumor repair systems, paracrine- or exosome-related signals have been reported to affect macrophage polarization and fibroblast activation, respectively, and to be associated with processes such as angiogenesis or tissue remodeling, and that previous *in vitro* GBM studies have also suggested that radiotherapy-modulated microglia can promote GBM cell proliferation and induce metabolic alterations through paracrine mechanisms, this study retained focused visualization of myeloid cells and fibroblasts in the cell-cell communication analysis and further emphasized the potential input relationships between myeloid cells and GSTCs in order to evaluate microenvironmental inputs as completely as possible, without presupposing that gliomas have mechanisms identical to those reported in the above studies (22–24). Given that local microenvironmental cues can jointly influence cellular behavior, whereas single-cell transcriptomic analysis, although helpful for resolving glioma cell state heterogeneity, does not preserve the original spatial positions of cells, the cell-cell communication results in this study should be understood as inferences of potential interactions based on ligand-receptor expression patterns rather than direct evidence of *in situ* cellular adjacency relationships (25, 26).

### 2.7 pySCENIC analysis

pySCENIC analysis was used to reconstruct gene regulatory networks from scRNA-seq data and to identify stable cell states. It should be noted that, in this study, the results generated by the pySCENIC module (v0.12.1) implemented in Python (v3.9.19) were visualized using R (v4.3.3), including the generation of an AUCell matrix to evaluate the enrichment levels of transcription factors and the activity of regulatory factors.

### 2.8 Cell culture and reagents

Human glioma cell lines LN229 and U251 were obtained from the Cell Bank of the Chinese Academy of Sciences (Shanghai, China). All cell lines were authenticated by short tandem repeat (STR) profiling and tested negative for mycoplasma contamination. Cells were cultured in Dulbecco’s Modified Eagle Medium supplemented with 10% fetal bovine serum (FBS) and 1% penicillin-streptomycin at 37 °C in a humidified incubator containing 5% CO_2_. LN229 and U251 were used in this study as conventional glioma cell-line models for preliminary functional assessment of CEBPD. We acknowledge that these established serum-cultured cell lines may not fully recapitulate the transcriptomic and phenotypic architecture of the computationally defined C0 VIM+ GSTC state. In particular, systematic molecular matching of the selected cell lines to the C0 signature, including IDH status and the expression levels of VIM, CHI3L1, VEGFA, and other state-associated markers, was not performed in the present study. Therefore, the cell-line experiments were designed to assess the phenotypic relevance of CEBPD in glioma cells, rather than to validate LN229 or U251 as complete *in vitro* equivalents of the C0 VIM+ GSTC population. Key reagents used in this study included the Cell Counting Kit-8 (CCK-8; Dojindo, Cat#CK04-500), Annexin V-FITC/PI apoptosis detection kit (BD Biosciences, Cat#556547), crystal violet (Sigma-Aldrich, Cat#C0775), and TRIzol reagent (Invitrogen, Cat#15596018) for total RNA isolation.

### 2.9 siRNA-mediated knockdown of CEBPD

To investigate the biological role of CEBPD in glioma cells, transient knockdown of CEBPD was achieved using two independent small interfering RNAs (siRNAs). U251 and LN229 cells were seeded in 6-well plates and transfected with siRNAs targeting CEBPD (si-CEBPD#1 and si-CEBPD#2) or a non-targeting negative control siRNA (si-Ctrl) using Lipofectamine 3000 (Invitrogen, Cat#L3000-015) according to the manufacturer’s instructions. The final siRNA concentration was 50 nM unless otherwise indicated. After 6 h of incubation, the culture medium was replaced with fresh complete medium, and cells were further cultured for 24–48 h before subsequent analyses.

Knockdown efficiency was evaluated by quantitative real-time PCR (qRT-PCR) at 24 h after transfection. Cells with validated CEBPD silencing were then subjected to downstream functional assays, including CCK-8 assay, colony formation assay, Transwell migration assay, and apoptosis analysis by flow cytometry. The sequences of si-CEBPD#1 and si-CEBPD#2, together with information on the non-targeting control siRNA (si-Ctrl), are provided in **Supplementary Table 2**.

### 2.10 Quantitative real-time PCR

Total RNA was extracted using TRIzol reagent following the manufacturer’s protocol, and cDNA was synthesized with the PrimeScript RT reagent kit (Takara, Cat#RR037A). qRT-PCR was performed using TB Green Premix Ex Taq II (Takara, Cat#RR820A) on a CFX96 Real-Time PCR Detection System (Bio-Rad, USA). Primer sequences are provided in **Supplementary Table 2**.

Relative expression was calculated using the 2^–ΔΔCt^ method, with β-actin serving as the internal control.

### 2.11 Cell proliferation assay

Cells (2 × 10³ per well) were seeded in 96-well plates in triplicate. At 0, 1, 2, 3, and 4 days after seeding, 10 µL of CCK-8 reagent was added to each well and incubated for 2 h at 37 °C. Absorbance was measured at 450 nm using a BioTek Synergy HTX microplate reader. The optical density (OD450) value was directly proportional to viable cell number, reflecting proliferation kinetics.

### 2.12 Colony formation assay

For assessment of long-term clonogenic survival, LN229 and U251 cells were seeded at 500–1000 cells/well in 6-well plates and cultured for 10–14 days until visible colonies appeared. Cells were fixed with 4% paraformaldehyde for 15 min and stained with 0.1% crystal violet for 20 min. Colonies containing more than 50 cells were counted under a stereomicroscope, and relative colony numbers were quantified using ImageJ.

### 2.13 Transwell migration assays

For the Transwell assay, 2 × 10^4^ cells suspended in serum-free medium were seeded in the upper chamber of Transwell inserts (8 µm pore size; Corning, Cat#3422), while medium supplemented with 10% FBS was placed in the lower chamber as a chemoattractant. After 24 h, migrated cells on the lower membrane surface were fixed, stained with 0.1% crystal violet, and counted in five random fields under 200× magnification.

### 2.14 Apoptosis analysis by flow cytometry

Apoptosis was analyzed using the Annexin V-FITC/PI Apoptosis Detection Kit (BD Biosciences, Cat#556547). Cells (1 × 10^6^) were harvested, washed twice with cold phosphate-buffered saline (PBS), and resuspended in 100 µL of binding buffer containing 5 µL Annexin V-FITC and 5 µL PI. After incubation for 15 min in the dark at room temperature, fluorescence was detected on a BD FACSCanto II flow cytometer. Data were analyzed using FlowJo v10, and both early (Annexin V^+^/PI^-^) and late (Annexin V^+^/PI^+^) apoptotic fractions were quantified.

### 2.15 Statistical analysis

All experiments were independently repeated at least three times. Data are presented as mean ± standard deviation (SD). Statistical significance was evaluated using a two-tailed Student’s t-test for two-group comparisons or one-way analysis of variance (ANOVA) followed by Tukey’s *post hoc* test for multiple comparisons, using GraphPad Prism 9.0 software. A P value < 0.05 was considered statistically significant.

## 3 Results

### 3.1 Construction of a single-cell atlas of gliomas across different IDH molecular backgrounds reveals state heterogeneity of GSTCs

**Figure 1:**
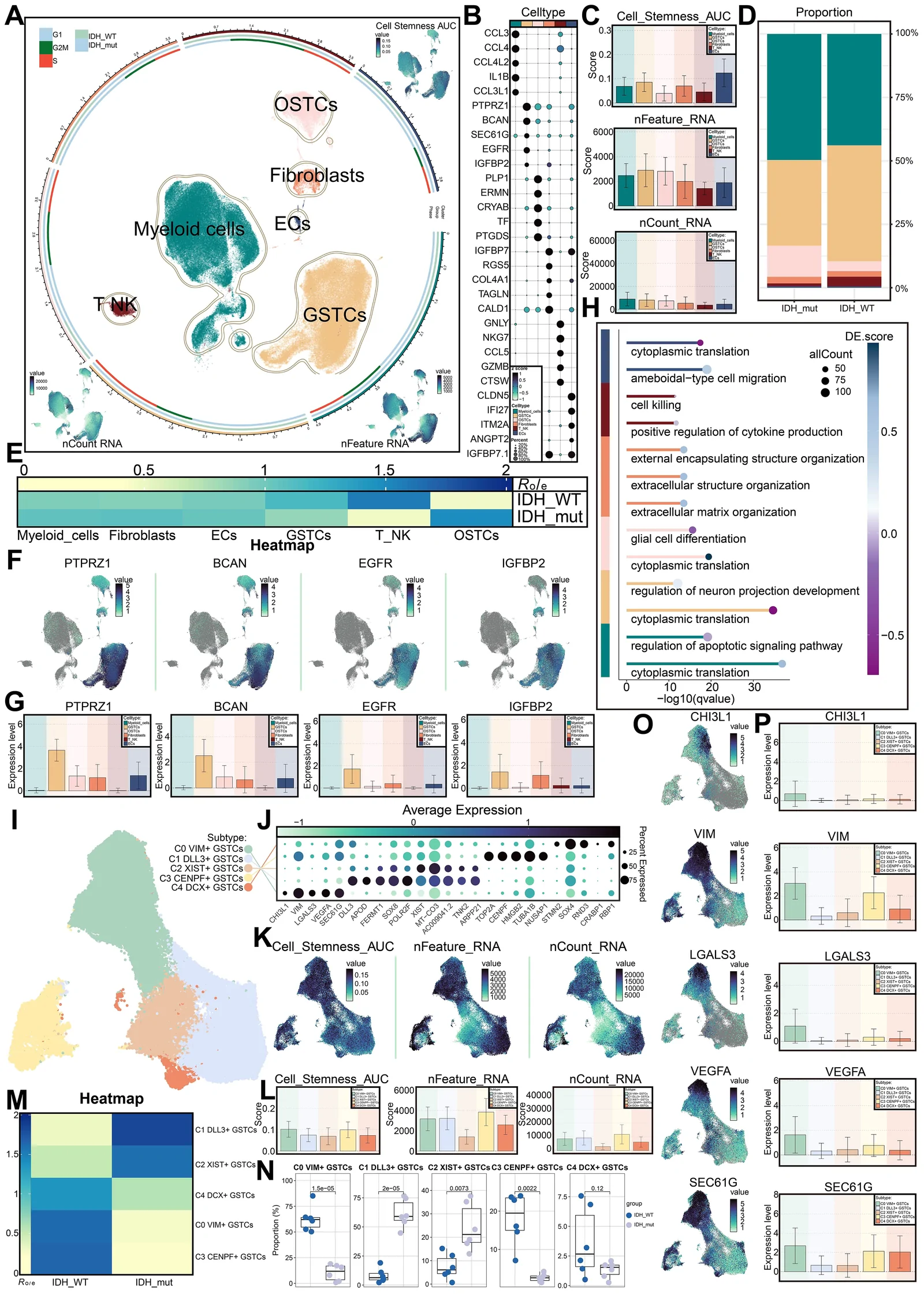
Construction of a single-cell atlas of gliomas across different IDH molecular backgrounds and state stratification of GSTCs. **(A)** Single-cell UMAP atlas of integrated IDH-WT and IDH-mut glioma samples, showing six major cell populations: myeloid cells, GSTCs, OSTCs, fibroblasts, T/NK cells, and ECs. The outer rings indicate cell-cycle states and sample origin (IDH-WT/IDH-mut), and display the distributions of Cell_Stemness_AUC, nFeature_RNA, and nCount_RNA. **(B)** Bubble plot showing representative marker genes of the major cell populations. **(C)** Comparison of Cell_Stemness_AUC, nFeature_RNA, and nCount_RNA among the major cell populations. **(D)** Stacked bar plot showing the proportions of the major cell populations in IDH-mut and IDH-WT samples. **(E)** Heatmap showing the relative enrichment of the major cell populations across the two IDH molecular backgrounds. **(F)** Feature plots of representative GSTC markers, including PTPRZ1, BCAN, EGFR, and IGFBP2. **(G)** Comparison of the expression levels of the above representative markers among the major cell populations. **(H)** Representative GO biological process enrichment results based on differentially expressed genes in each major cell population. **(I)** UMAP plot of reclustered GSTCs, identifying five subgroups: C0 VIM+ GSTCs, C1 DLL3+ GSTCs, C2 XIST+ GSTCs, C3 CENPF+ GSTCs, and C4 DCX+ GSTCs. **(J)** Bubble plot showing marker genes of the five GSTC subgroups. **(K)** Distribution of Cell_Stemness_AUC, nFeature_RNA, and nCount_RNA within GSTCs. **(L)** Comparison of Cell_Stemness_AUC, nFeature_RNA, and nCount_RNA among the GSTC subgroups. **(M)** Heatmap showing the distribution tendencies of the five GSTC subgroups in IDH-WT and IDH-mut samples. **(N)** Comparison of the proportions of GSTC subgroups across different IDH molecular backgrounds. **(O)** Feature plots of representative markers of C0 VIM+ GSTCs, including CHI3L1, VIM, LGALS3, VEGFA, and SEC61G. **(P)** Comparison of the expression levels of the above representative markers among the five GSTC subgroups.

Based on the publicly available single-cell transcriptomic dataset GSE278456, we performed an integrated discovery-oriented analysis of glioma samples with different IDH molecular backgrounds to evaluate tumor cell state heterogeneity and its relationship with the microenvironment. This dataset was derived from a single-cell study of 33 glioma cases, providing a unified analytical basis for simultaneously observing malignant tumor cells and their associated microenvironmental populations, including the myeloid niche (27). After dimension reduction and clustering of all cells, a total of 35 cell clusters were identified. Based on the original literature of data GSE278456 and known marker genes, combined with the inferCNV analysis used to assist in identifying malignant cell populations, we discovered two cell types: GSTCs and oligodendrocyte-like stem tumor cells (OSTCs) (**Supplementary Figures 1A–F**). All cells were further categorized into six major populations, including myeloid cells, GSTCs, OSTCs, fibroblasts, T cells and natural killer cells (T/NK cells), and endothelial cells (ECs) (**Figure 1A**). The outer rings in **Figure 1A** also display cell-cycle states, the distribution of IDH-WT/IDH-mut origins, and the variation of Cell_Stemness_AUC, nFeature_RNA, and nCount_RNA across all cells.

The representative marker genes of each major cell population further supported the above annotations. Myeloid cells mainly expressed CCL3, CCL4, CCL4L2, IL1B, and CCL3L1; GSTCs highly expressed PTPRZ1, BCAN, SEC61G, EGFR, and IGFBP2; OSTCs were enriched for PLP1, ERMN, CRYAB, TF, and PTGDS; fibroblasts mainly expressed IGFBP7, RGS5, COL4A1, TAGLN, and CALD1; T/NK cells were represented by GNLY, NKG7, CCL5, GZMB, and CTSW; and ECs mainly expressed CLDN5, IFI27, ITM2A, ANGPT2, and IGFBP7 (**Figure 1B**). Further comparison of Cell_Stemness_AUC, nFeature_RNA, and nCount_RNA across different cell populations showed clear differences among the groups, with myeloid cells and GSTCs generally exhibiting relatively higher levels, suggesting substantial heterogeneity among different cell populations in stemness-related features and transcriptional activity (**Figure 1C**).

At the level of cellular composition, the comparison between the IDH wild-type (IDH-WT) and IDH-mutant (IDH-mut) groups was conducted using individual patient samples as the statistical unit, rather than individual cells. Firstly, the cells of each patient were aggregated at the sample level for corresponding analyses; subsequently, all statistical comparisons between the two groups were based on patient-level data. Both IDH-mut and IDH-WT samples were mainly composed of myeloid cells and GSTCs, but their internal composition showed distinct distribution patterns. Compared with the IDH-mut group, the IDH-WT group showed relatively higher proportions of GSTCs and T/NK cells, whereas OSTCs were relatively more abundant in the IDH-mut group (**Figures 1D, E**). For GSTCs, we further selected PTPRZ1, BCAN, EGFR, and IGFBP2 for visualization and quantitative comparison. The results showed that these molecules were mainly concentrated in the GSTC region, among which PTPRZ1, EGFR, and IGFBP2 were particularly prominent in GSTCs, while BCAN also maintained relatively high expression in this population (**Figure 1F**). The quantitative results were largely consistent with the UMAP feature plots, further supporting the identification of GSTCs as the core malignant tumor cell population (**Figure 1G**).

To summarize the different cell populations at the functional level, we performed GO enrichment analysis of the differentially expressed genes in each population. The results showed that both myeloid cells and GSTCs were associated with terms such as cytoplasmic translation and regulation of apoptotic signaling pathway; OSTCs were mainly associated with glial cell differentiation; fibroblasts were significantly enriched in external encapsulating structure organization, external structure organization, and extracellular matrix organization; T/NK cells were mainly enriched in cell killing and positive regulation of cytokine production; and ECs were more involved in amoeboidal-type cell migration and cytoplasmic translation (**Figure 1H**). Overall, the marker expression patterns of each cell population were highly consistent with their functional enrichment results.

On this basis, we further reclustered GSTCs to resolve their internal state heterogeneity. A total of five GSTC subgroups were identified, designated as C0 VIM+ GSTCs, C1 DLL3+ GSTCs, C2 XIST+ GSTCs, C3 CENPF+ GSTCs, and C4 DCX+ GSTCs (**Figure 1I**). The marker genes of each subgroup displayed clear state stratification: C0 was represented by CHI3L1, VIM, LGALS3, VEGFA, and SEC61G; C1 mainly expressed DLL3, APOD, FERMT1, SOX8, and POLR2F; C2 prominently expressed XIST, MT-CO3, AC009041.2, TNIK, and ARPP21; C3 was enriched for cell cycle-related genes such as TOP2A, CENPF, HMGB2, TUBA1B, and NUSAP1; and C4 mainly expressed neural differentiation-related molecules such as STMN2, SOX4, RND3, CRABP1, and RBP1 (**Figure 1J**). Further comparison of Cell_Stemness_AUC, nFeature_RNA, and nCount_RNA among the subgroups revealed marked state differences within GSTCs, among which C3 CENPF+ GSTCs exhibited higher transcriptional complexity and transcriptional activity, consistent with their molecular features dominated by cell cycle-related genes (**Figures 1K, L**).

Subsequently, we analyzed the distribution characteristics of GSTC subgroups across different IDH molecular backgrounds. The heatmap showed that IDH-WT and IDH-mut differed in their relative compositional patterns across the five GSTC subgroups (**Figure 1M**). Further comparison of the proportions of each subgroup between the two groups showed that C0 VIM+ GSTCs and C3 CENPF+ GSTCs were relatively higher in the IDH-WT group, whereas C1 DLL3+ GSTCs and C2 XIST+ GSTCs were relatively more abundant in the IDH-mut group; C4 DCX+ GSTCs showed no obvious difference between the two groups (**Figure 1N**). Further visualization and quantitative analysis of representative genes in C0 showed that the expression of CHI3L1, VIM, LGALS3, VEGFA, and SEC61G was mainly concentrated in the UMAP region corresponding to C0 and that the overall expression levels were highest or relatively high in C0 (**Figures 1O, P**). In summary, GSTCs are not a homogeneous population, but rather consist of multiple cell states with distinct molecular features and differential distribution tendencies across IDH backgrounds. Among them, C0 VIM+ GSTCs are relatively enriched in IDH-WT and exhibit more distinct molecular features; therefore, this subgroup was selected as the focus of subsequent analyses.

### 3.2 Functional profiling shows that C0 VIM+ GSTCs exhibit composite features related to mesenchymal-like properties and microenvironmental adaptation

**Figure 2:**
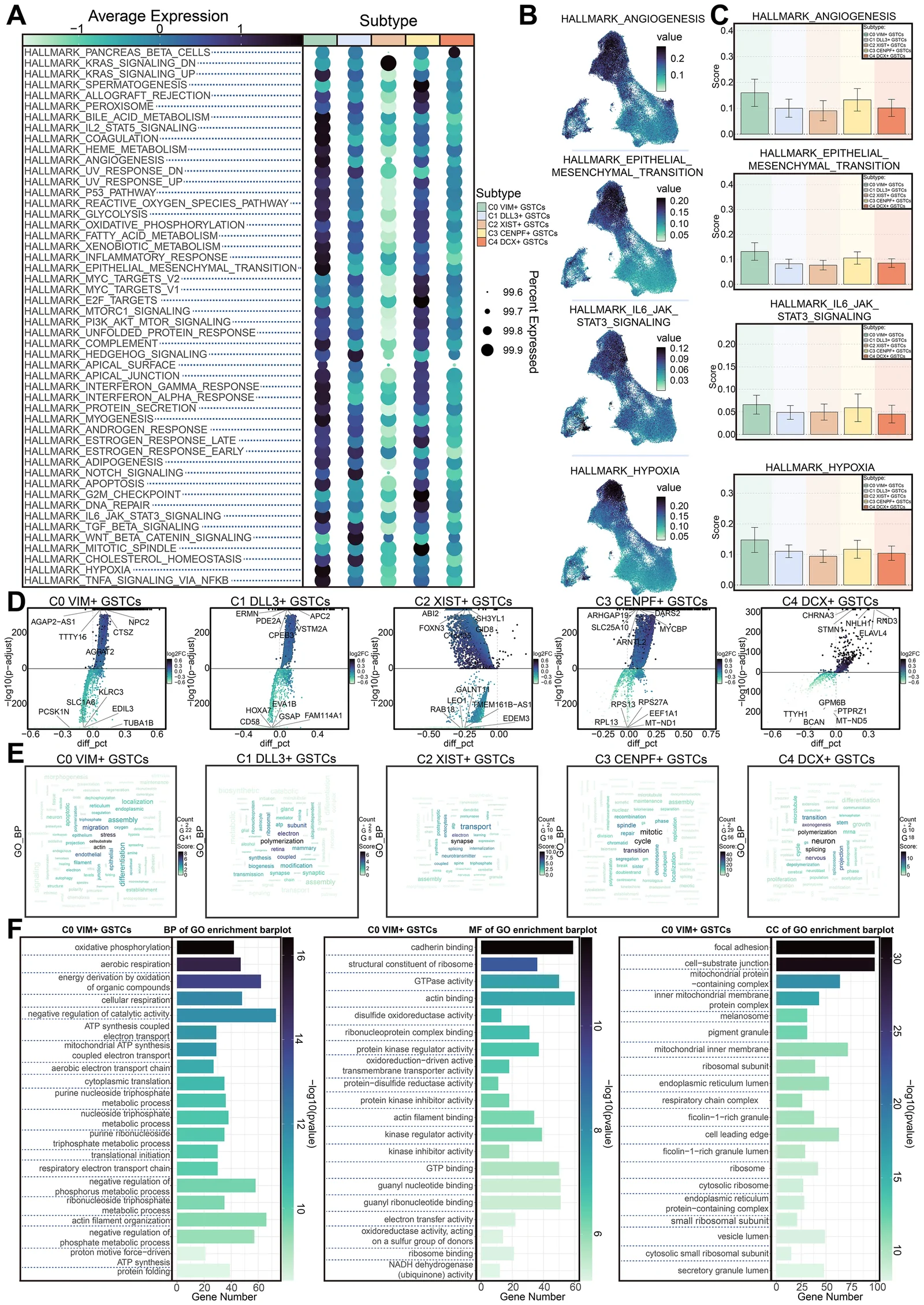
Functional profiling indicates that C0 VIM+ GSTCs display more prominent composite functional features. **(A)** Bubble plot of Hallmark pathway activities across the five GSTC subgroups, showing functional stratification among different states. **(B)** UMAP projections of representative pathways, including HALLMARK_ANGIOGENESIS, HALLMARK_EPITHELIAL_MESENCHYMAL_TRANSITION, HALLMARK_IL6_JAK_STAT3_SIGNALING, and HALLMARK_HYPOXIA. **(C)** Comparison of the scores of the above four representative pathways among the GSTC subgroups. **(D)** Volcano plots of differentially expressed genes for each of the five GSTC subgroups relative to the remaining subgroups. **(E)** GO biological process enrichment word clouds for each GSTC subgroup. **(F)** GO enrichment bar plots of differentially expressed genes in C0 VIM+ GSTCs, showing representative enriched terms at the levels of biological process (BP), molecular function (MF), and cellular component (CC).

To further compare the biological characteristics of different GSTC states, we performed a systematic functional profiling analysis of the five subgroups. Hallmark pathway analysis showed relatively clear functional stratification among the different GSTC subgroups. Compared with the other subgroups, C0 VIM+ GSTCs were overall more active in pathways such as angiogenesis, epithelial-mesenchymal transition, IL6/JAK/STAT3 signaling, and hypoxia, whereas C3 CENPF+ GSTCs were more inclined toward typical proliferation-related programs such as E2F targets, G2M checkpoint, DNA repair, and mitotic spindle (**Figure 2A**). In the UMAP projection, the high-scoring regions of the above representative pathways mainly corresponded to the region occupied by C0 (**Figure 2B**), and quantitative results further showed that C0 ranked highest or relatively high in angiogenesis, epithelial-mesenchymal transition, IL6/JAK/STAT3 signaling, and hypoxia scores (**Figure 2C**). These results suggest that C0 is not simply defined by high VIM expression, but more likely represents a composite cellular state characterized by inflammatory signaling response, hypoxic adaptation, mesenchymal-like transformation, and angiogenic remodeling.

Differential expression analysis of the different subgroups further supported the above state classification. The volcano plots showed that the five GSTC subgroups each had relatively independent differential expression profiles. In C0 VIM+ GSTCs, genes such as AGAP2-AS1, TTYH1, CTSZ, and NPC2 were more prominent; C1 DLL3+ GSTCs were represented by ERMN, PDE2A, APC2, and STMN2; C2 XIST+ GSTCs more prominently expressed ABI2, FOXN3, SH3YL1, and GNB5; in C3 CENPF+ GSTCs, genes such as ARHGAP19, SLC25A10, ARNTL2, DARS2, and MYCBP were more significant, which, together with the aforementioned transcriptional features, suggests that this subgroup is closer to an actively proliferative state; and C4 DCX+ GSTCs mainly expressed neural-related molecules such as CHRNA3, STMN2, NHLH1, ELAVL4, and RND3 (**Figure 2D**). The corresponding GO word cloud analysis was largely consistent with the above results: C3 was more biased toward processes such as mitosis, cell cycle, spindle, and transition; C4 showed more terms related to neuron, projection, growth, differentiation, and polymerization; whereas C0 was relatively more associated with functional features such as migration, assembly, stress, localization, and actin (**Figure 2E**).

Considering that C0 showed strong VIM-related expression features in the preceding analyses and was functionally more associated with migration, stress, and structural remodeling processes, we further performed a more in-depth GO enrichment analysis of the C0 subgroup. The results showed that, at the biological process level, C0 VIM+ GSTCs were mainly enriched in oxidative phosphorylation, aerobic respiration, ATP synthesis coupled electron transport, and cellular respiration; at the molecular function level, they mainly involved cadherin binding, GTPase activity, and actin binding; and at the cellular component level, they were mainly enriched in structures such as focal adhesion, cell-substrate junction, mitochondrial protein-containing complex, respiratory chain complex, and ribosome (**Figure 2F**). Taken together, these results indicate that C0 is more consistent with a GSTC state characterized by mesenchymal-like transformation, microenvironmental adaptation, and enhanced cell adhesion and cytoskeletal remodeling, rather than a tumor cell subgroup driven solely by proliferation. Because mitochondrial and respiration-related programs may partially overlap with cellular stress responses, this interpretation was not based solely on oxidative phosphorylation or respiratory-chain enrichment, but was integrated with non-mitochondrial C0 markers, IDH-associated distribution, trajectory position, communication inference, and regulon-level evidence.

### 3.3 Developmental trajectory and CellChat-based communication inference suggest that C0 is located at a state transition node and may receive candidate myeloid-derived OSM input

**Figure 3:**
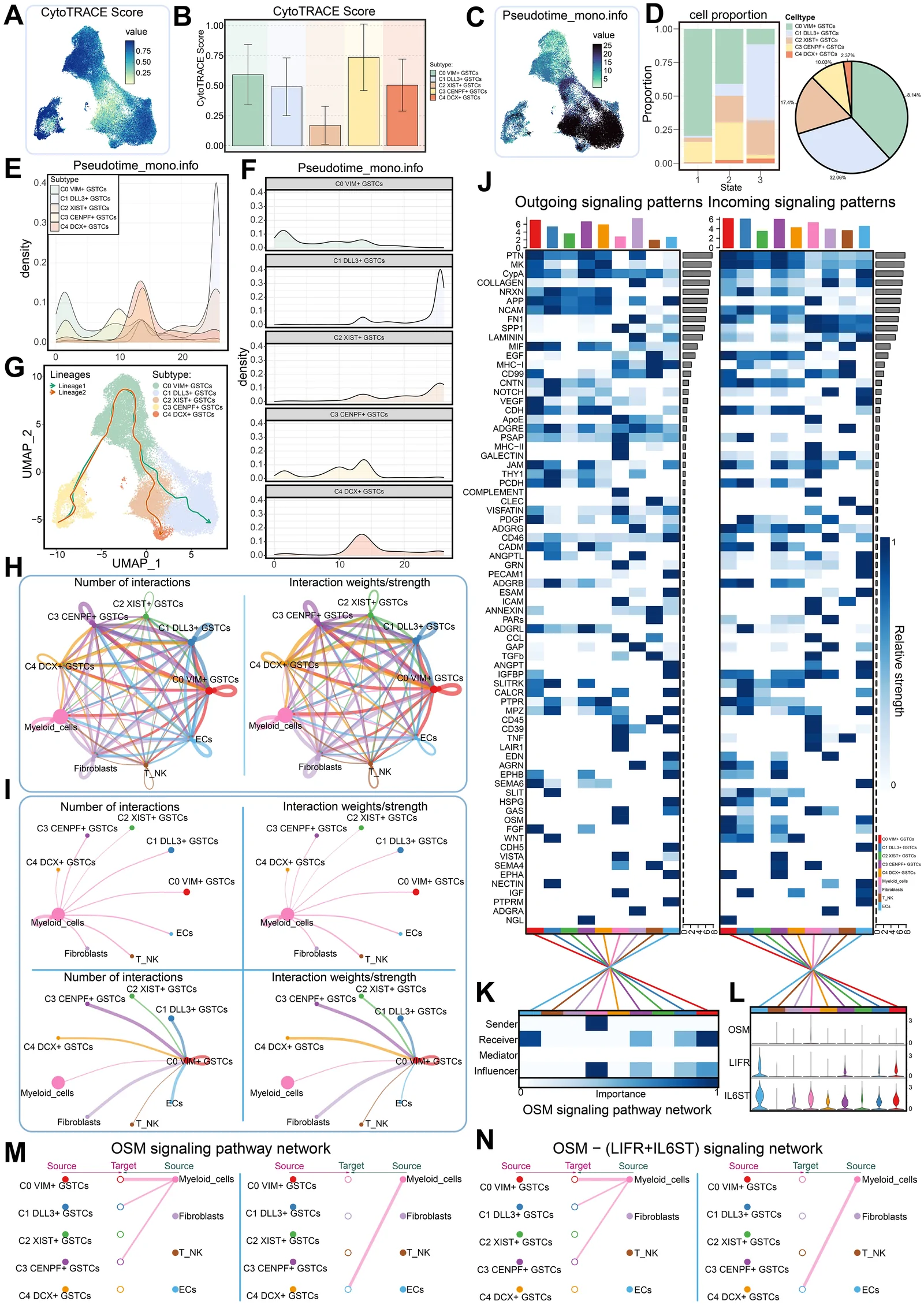
Developmental trajectory and CellChat-based communication inference nominate candidate myeloid-derived OSM input to C0 VIM+ GSTCs. **(A)** UMAP projection of CytoTRACE scores in GSTCs. **(B)** Comparison of CytoTRACE scores among the five GSTC subgroups. **(C)** Pseudotime trajectory of GSTCs reconstructed using Monocle. **(D)** Stacked bar plot showing the composition of GSTC subgroups across different pseudotime states, together with a pie chart showing the overall proportions of the five GSTC subgroups. **(E)** Density distribution of the five GSTC subgroups along pseudotime. **(F)** Faceted density plots showing the pseudotime distributions of individual GSTC subgroups. **(G)** Slingshot trajectory analysis showing the branched state-transition paths of GSTCs. **(H)** Global interaction networks between GSTC subgroups and microenvironmental cell populations, showing both the number and strength of interactions. **(I)** Interaction networks centered on myeloid cells and C0 VIM+ GSTCs, showing both the number and strength of interactions. **(J)** Heatmaps of outgoing and incoming signaling patterns across different cell populations. **(K)** Relative importance of different cell populations as senders, receivers, mediators, and influencers in the OSM signaling pathway network. **(L)** Expression distributions of OSM and the receptor complex-related molecules LIFR and IL6ST across different cell populations. **(M)** OSM signaling pathway network showing OSM-mediated communication among different cell populations. **(N)** OSM-(LIFR+IL6ST) signaling network showing OSM-mediated communication through the LIFR/IL6ST receptor complex.

After defining the composite functional features of C0 VIM+ GSTCs, we further evaluated their relative position within the GSTC state lineage. CytoTRACE analysis showed that the developmental potential within GSTCs was not uniform (**Figure 3A**). Among them, C3 CENPF+ GSTCs had the highest score, followed by C0 VIM+ GSTCs, whereas C2 XIST+ GSTCs had the lowest relative score (**Figure 3B**). These results suggest that C3 is closer to a highly stem-like and incompletely differentiated state; in contrast, although C0 retains relatively high plasticity, it is already accompanied by more evident functional output features.

Pseudotime reconstruction based on Monocle showed that GSTCs were not composed of mutually discrete subgroups, but rather exhibited a continuous process of state change (**Figure 3C**). After stratifying cells according to state, clear differences in cellular composition were observed among different states (**Figure 3D**). Overall, C0 VIM+ GSTCs and C1 DLL3+ GSTCs accounted for 38.14% and 32.06% of GSTCs, respectively, representing the two most abundant subgroups; C2 XIST+ GSTCs, C3 CENPF+ GSTCs, and C4 DCX+ GSTCs accounted for 17.40%, 10.03%, and 2.37%, respectively. In conjunction with their positions along the trajectory, State 1, State 2, and State 3 showed a relative ordering from early to late along pseudotime, with State 1 being mainly composed of C0. As pseudotime progressed, the proportions of C1 DLL3+ GSTCs and a subset of C2 XIST+ GSTCs gradually increased in States 2 and 3, suggesting that C0 was preferentially distributed in the early segment of pseudotime and connected to subsequent state patterns dominated by C1 and a subset of C2. Pseudotime density distribution analysis further supported this trend: C0 VIM+ GSTCs and C3 CENPF+ GSTCs were mainly distributed in the early-to-middle segments of the trajectory, C4 DCX+ GSTCs were more often located in the middle segment, whereas C1 DLL3+ GSTCs and C2 XIST+ GSTCs were more often distributed in the later segment of the trajectory (**Figures 3E, F**).

Trajectory inference using Slingshot further revealed that GSTC state progression also exhibited branching features. Overall, the trajectory extended progressively from early-state cells to C0 VIM+ GSTCs and then further diverged into two directions leading to C1 DLL3+ GSTCs and C2 XIST+ GSTCs, respectively, with the branch toward C2 eventually extending further to C4 DCX+ GSTCs (**Figure 3G**). Although Monocle and Slingshot showed some differences in local trajectory structure, both supported branched progression of GSTCs on the basis of continuous state changes and jointly suggested that C0 occupies an important position linking early-state cells to subsequent branch transitions.

After clarifying the lineage position of C0, we further analyzed its cell-cell communication relationships with the tumor microenvironment. The overall interaction network showed that all GSTC subgroups had extensive connections with myeloid cells, ECs, fibroblasts, and T/NK cells, among which myeloid cells constituted an important communication hub in the microenvironment, while C0 VIM+ GSTCs were also among the more communication-active subgroups within GSTCs (**Figure 3H**). Further dissection of the network showed that myeloid cells maintained clear interactions with multiple GSTC subgroups and that the interactions between C0 and myeloid cells were relatively prominent in the bidirectional network (**Figure 3I**).

Based on the overall communication landscape described above, we further focused on potential key signaling axes between C0 VIM+ GSTCs and myeloid cells. Among the candidate pathways, OSM signaling exhibited a relatively clearer pattern (**Figure 3J**). In the OSM signaling pathway network, C0 VIM+ GSTCs mainly acted as receivers, whereas myeloid cells mainly participated in this communication process as senders and influencers (**Figure 3K**). The ligand-receptor expression pattern showed that OSM was mainly derived from myeloid cells; LIFR showed an overall low expression level, but was detectable in some cells; IL6ST had a broader expression basis, including in C0 VIM+ GSTCs (**Figure 3L**). Further dissection of the network showed that the predicted communication relationship from myeloid cells to C0 was retained both in the overall OSM signaling pathway network and when further restricted to the OSM-(LIFR+IL6ST) receptor complex (**Figures 3M, N**). These CellChat-based results suggest that myeloid cell-derived OSM signaling may constitute one of the candidate external inputs to C0 VIM+ GSTCs. However, because this inference is derived from ligand–receptor co-expression and network modeling, it should not be regarded as direct functional or spatial evidence. Whether OSM signaling directly participates in the formation or maintenance of the C0 state still requires further validation by co-culture experiments, ligand or receptor perturbation, and spatially resolved analyses. Notably, previous studies have suggested that, under radiotherapy conditions, paracrine signals derived from microglia can promote GBM cell proliferation together with metabolic changes (24). Although the context of that study is not entirely identical to that of the present study, its findings support, at the directional level, the possibility that myeloid/microglia-derived inputs may participate in the regulation of tumor cell states.

### 3.4 pySCENIC analysis reveals CEBPD-associated regulon features and M2 module preference related to the C0 state

**Figure 4:**
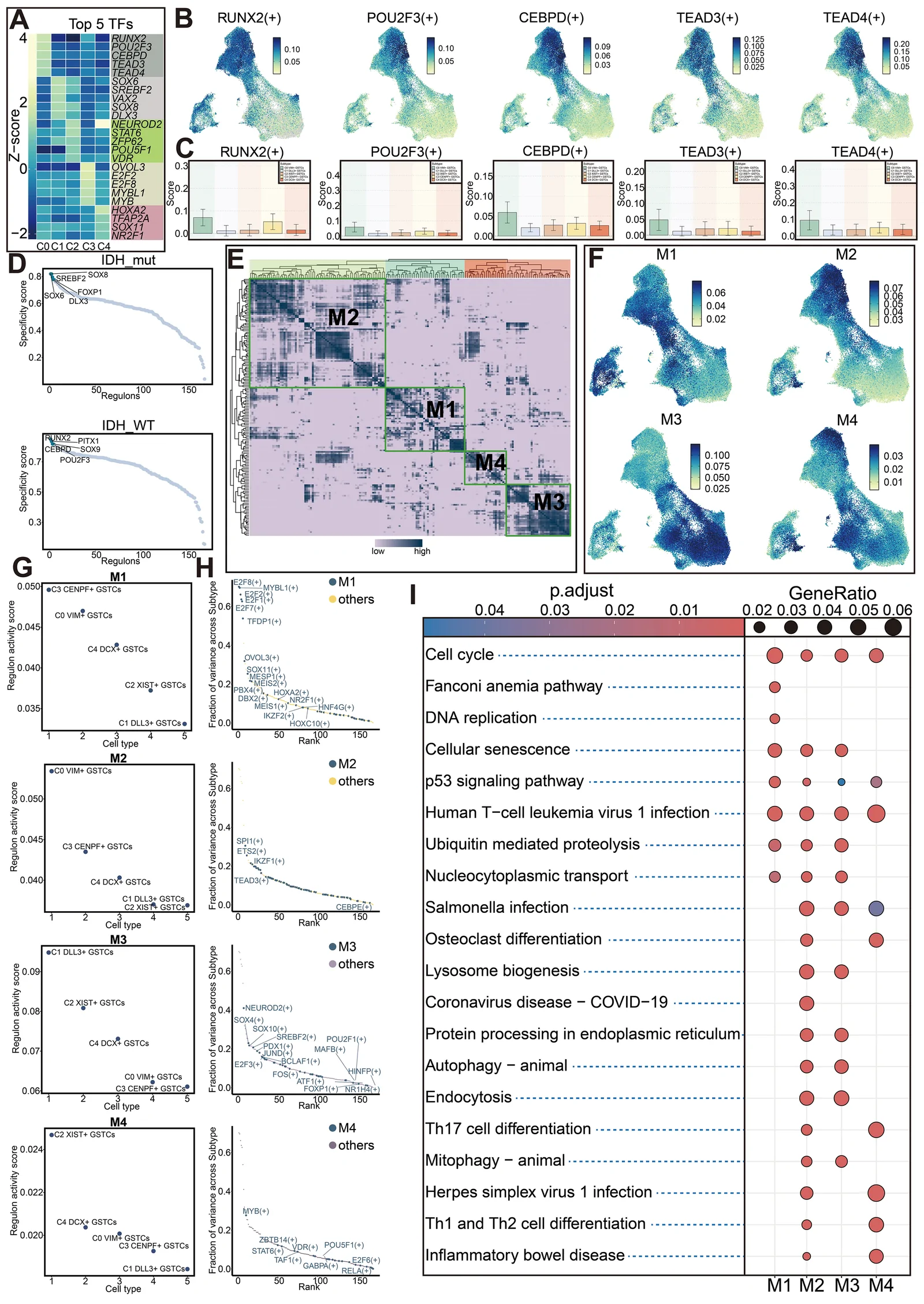
pySCENIC analysis identifies C0-associated regulon activity and nominates CEBPD as a candidate regulatory factor. **(A)** Heatmap showing the top five transcription factors/regulons across the five GSTC subgroups. **(B)** UMAP projections of RUNX2(+), POU2F3(+), CEBPD(+), TEAD3(+), and TEAD4(+) regulon activities. **(C)** Comparison of the activities of the above representative regulons among the five GSTC subgroups. **(D)** Ranking plots of regulon specificity scores calculated separately for the IDH-mut and IDH-WT molecular backgrounds. **(E)** Heatmap of regulon modules constructed based on co-activation patterns, identifying four transcriptional regulatory modules (M1–M4). **(F)** UMAP projections of M1–M4 module activities. **(G)** Distribution of regulon activity scores of the four modules across different GSTC subgroups. **(H)** Ranking of representative regulons in each module and the distribution of their variance contributions across different subgroups. **(I)** Bubble plot of pathway enrichment for the M1–M4 modules, showing representative functional terms associated with each module.

To further characterize the state features of C0 VIM+ GSTCs at the level of transcriptional regulation, we performed pySCENIC analysis on the five GSTC subgroups. Regulon activity across the different subgroups exhibited a relatively clear stratification pattern (**Figure 4A**). Among them, RUNX2, POU2F3, CEBPD, TEAD3, and TEAD4 were most prominent in C0 VIM+ GSTCs. UMAP projection further showed that the high-activity regions of these five regulons were mainly concentrated in the region corresponding to C0 (**Figure 4B**), and quantitative results also indicated that C0 ranked highest or relatively high in the scores of RUNX2(+), POU2F3(+), CEBPD(+), TEAD3(+), and TEAD4(+) (**Figure 4C**). These results indicate that C0 is distinguished from other GSTC subgroups not only at the level of marker genes, but also by relatively systematic activation of transcriptional regulatory programs. Based on its relatively prominent activity in C0, CEBPD was selected as a candidate regulatory factor for subsequent focused investigation.

Further comparison of regulon specificity according to IDH molecular background made this regulatory difference more evident. In IDH-mut samples, the regulons with relatively higher specificity mainly included SREBF2, SOX8, SOX6, FOXP1, and DLX3; in contrast, RUNX2, PITX1, CEBPD, SOX9, and POU2F3 were relatively more prominent in IDH-WT samples (**Figure 4D**). This result is consistent with the previous observation that C0 VIM+ GSTCs are relatively enriched in IDH-WT, suggesting that differences in IDH-WT-associated malignant states are reflected not only at the level of cellular composition, but also in the remodeling of transcriptional regulatory features.

To further reduce the complexity of the regulon network, we grouped all regulons into four transcriptional regulatory modules (M1–M4) according to co-activation patterns (**Figure 4E**). The distribution of these modules in the GSTC atlas showed relatively clear state preferences (**Figure 4F**). Among them, M2 was the module most strongly concentrated in C0 VIM+ GSTCs; M1 was mainly biased toward C3 CENPF+ GSTCs, M3 was most active in C1 DLL3+ GSTCs, and M4 was mainly enriched in C2 XIST+ GSTCs; C4 DCX+ GSTCs did not show obvious dominance by a single module, but instead showed a certain degree of activity in both M3 and M4 (**Figure 4G**). This result suggests that, at the module level, the correspondence between M2 and C0 is the most evident.

In terms of representative regulon composition, M1 was mainly composed of cell cycle-related factors such as E2F1, E2F2, E2F7, E2F8, MYBL1, and TFDP1; M2 included transcription factors such as SPI1, ETS2, IKZF1, TEAD3, and CEBPE; M3 involved more regulators associated with neural development and state transition, including NEUROD2, SOX4, SOX10, SREBF2, and POU2F1; and M4 was represented by factors such as MYB, STAT6, VDR, POU5F1, GABPA, and RELA (**Figure 4H**). Overall, M1 displayed typical proliferation-related regulatory features, whereas M2 reflected another type of regulatory pattern distinct from cell cycle programs.

Further functional enrichment analysis of the four modules showed that M1 was mainly enriched in terms such as cell cycle, Fanconi anemia pathway, DNA replication, cellular senescence, p53 signaling pathway, ubiquitin mediated proteolysis, and nucleocytoplasmic transport, suggesting that it is most closely related to typical proliferation- and replication stress-associated programs. In contrast, in addition to involving some cell cycle, cellular senescence, and p53 signaling pathway terms, M2 was further enriched in ubiquitin mediated proteolysis, nucleocytoplasmic transport, lysosome biogenesis, protein processing in endoplasmic reticulum, autophagy-animal, endocytosis, and mitophagy-animal (**Figure 4I**). These enrichment features suggest that M2 is not dominated by a single pathway, but is more consistent with a non-proliferative regulatory program based on the maintenance of protein homeostasis, intracellular transport, membrane system remodeling, and stress adaptation. Given its strong correspondence with C0 VIM+ GSTCs, M2 may constitute an important regulatory basis for the C0-related state. Overall, pySCENIC analysis further supports the existence of C0 as a relatively independent GSTC state at the level of transcriptional regulation and provides a basis for subsequent functional validation centered on CEBPD.

### 3.5 CEBPD knockdown inhibits growth- and migration-related phenotypes while increasing apoptosis in glioma cells

Before interpreting these functional assays, we emphasize that LN229 and U251 are conventional glioma cell lines and are not equivalent to patient-derived or sorted C0 VIM+ GSTCs. These experiments were therefore designed to examine whether CEBPD, a candidate regulator nominated by the single-cell regulon analysis, contributes to malignant phenotypes in glioma cells. They should be interpreted as target-level functional support for CEBPD rather than as direct validation of the complete C0 VIM+ GSTC state.

**Figure 5:**
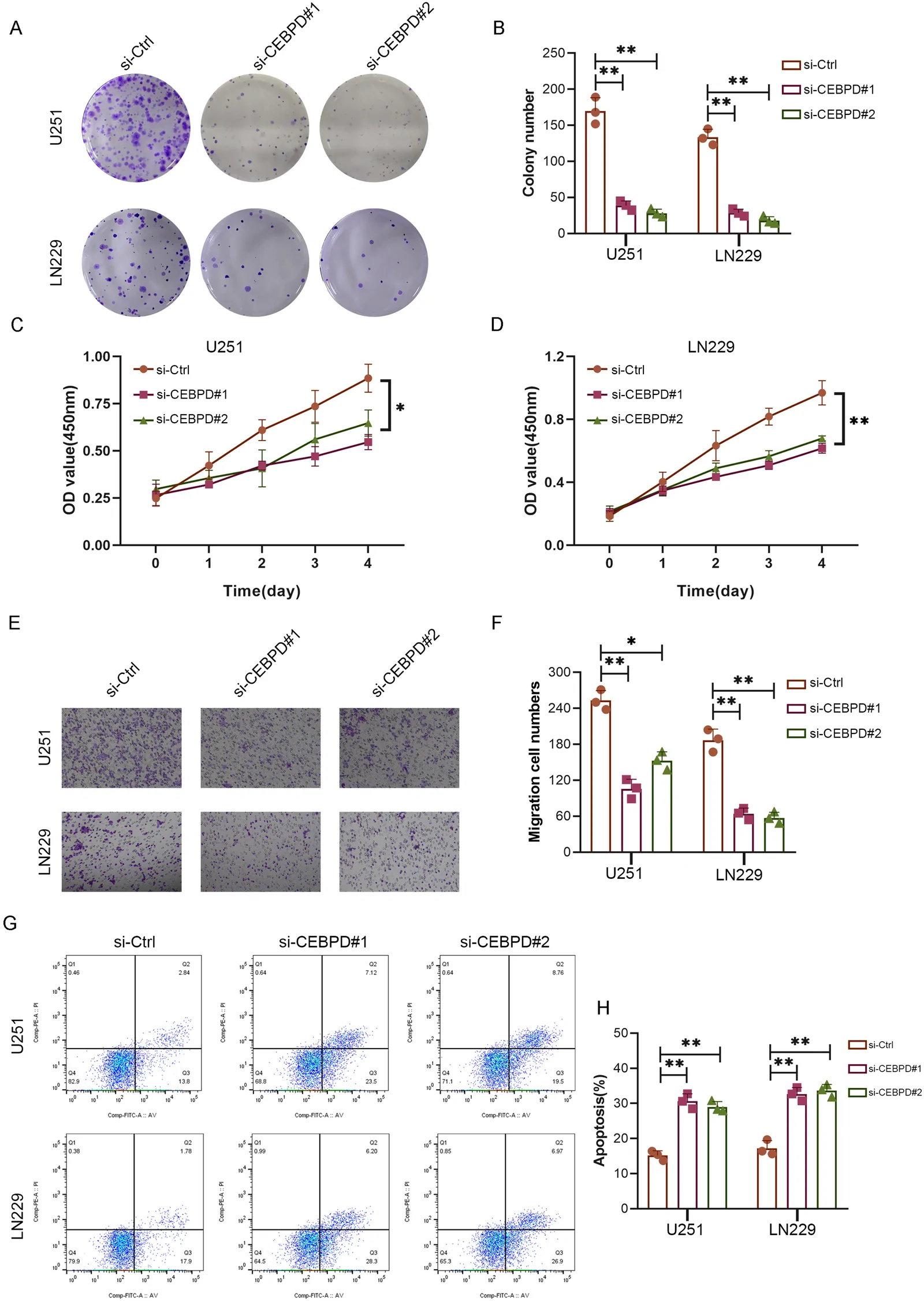
CEBPD knockdown impairs glioma cell growth and migration and increases apoptosis. **(A)** Colony formation assays in U251 and LN229 cells transfected with si-Ctrl, si-CEBPD#1, or si-CEBPD#2. **(B)** Quantification of colony numbers in the indicated groups. **(C–D)** CCK-8 growth curves of U251 **(C)** and LN229 **(D)** cells after CEBPD silencing. **(E)** Transwell migration assays in U251 and LN229 cells transfected with si-Ctrl, si-CEBPD#1, or si-CEBPD#2. **(F)** Quantification of migrated cell numbers in the indicated groups. **(G)** Annexin V-FITC/PI flow cytometric plots of U251 and LN229 cells following CEBPD knockdown. **(H)** Quantification of apoptotic cell percentages in the indicated groups. Data are presented as mean ± SD. *P < 0.05, **P < 0.01.

After transfection with two independent siRNAs targeting CEBPD, U251 and LN229 cells were subjected to colony formation, CCK-8, Transwell migration, and apoptosis assays. In the colony formation assay, both si-CEBPD#1 and si-CEBPD#2 reduced the colony-forming ability of the two cell lines. Compared with the si-Ctrl group, the numbers of colonies were obviously decreased after CEBPD silencing, and the colonies that remained were generally smaller (**Figures 5A, B**), suggesting that CEBPD is involved in maintaining clonogenic growth.

The CCK-8 results showed a similar tendency. Although OD450 values increased gradually in all groups over time, the increase in the si-CEBPD groups was weaker than that in the control group in both U251 and LN229 cells, and the difference became more apparent at the later time points (**Figures 5C, D**). These findings suggest that CEBPD knockdown suppresses glioma cell growth.

We next examined the effect of CEBPD knockdown on cell migration. In Transwell assays, fewer cells migrated through the membrane in the si-CEBPD groups than in the si-Ctrl group in both cell lines (**Figures 5E, F**), indicating that CEBPD silencing was accompanied by reduced migratory activity.

Apoptosis analysis showed an opposite pattern. Annexin V-FITC/PI staining revealed that the proportion of apoptotic cells was higher in the two knockdown groups than in the control group in both U251 and LN229 cells (**Figures 5G, H**), suggesting that reduced CEBPD expression compromises glioma cell survival.

The efficiency of knockdown was further examined by qRT-PCR. In both cell lines, CEBPD mRNA levels in the si-CEBPD#1 and si-CEBPD#2 groups were clearly lower than those in the si-Ctrl group (**Supplementary Figure 2**), confirming effective silencing of CEBPD under the present experimental conditions.

Taken together, these results show that CEBPD knockdown weakens colony formation, cell growth, and migration, while increasing apoptosis in conventional glioma cell-line models. These findings support the involvement of CEBPD in malignant phenotypes in glioma cells, while not by themselves establishing CEBPD as a fully validated master regulator of the patient-derived C0 VIM+ GSTC state.

## 4 Discussion

This study systematically delineated the cellular composition of gliomas across different IDH molecular backgrounds, resolved the internal state heterogeneity of GSTCs, and further performed a target-level functional assessment of a candidate regulatory factor through complementary *in vitro* experiments. Rather than merely cataloging compositional differences, the study specifically sought to determine whether an IDH-WT-associated GSTC state exists that integrates invasive potential with microenvironmental adaptation, and whether this state can be linked to extrinsic myeloid-derived inputs and intrinsic regulatory programs. Overall, our findings support a framework in which C0 VIM+ GSTCs represent a relatively IDH-WT-enriched malignant state marked by mesenchymal-like, hypoxia-adaptive, angiogenesis-related, and inflammatory features, thereby linking this state to a vascular–immune microenvironmental context and nominating CEBPD as a candidate regulatory node.

In the overall single-cell atlas, myeloid cells and GSTCs constituted the major components of the tumor tissue, supporting the view that glioma state progression is shaped not only by tumor cell-intrinsic programs but also by sustained interactions between malignant states and a myeloid-enriched microenvironment. We observed enrichment of inflammation-related molecules such as CCL3, CCL4, and IL1B in myeloid cells, and previous studies have linked these molecules to protumor neutrophil activation, cell migration, and EMT-related phenotypes, respectively (28–30). At the same time, other single-cell studies have also suggested that the immunosuppressive niche is often shaped jointly by multiple myeloid and stromal components rather than by a single immune cell subgroup; the limited efficacy of immunotherapy in glioma is likewise closely related to the infiltration of suppressive myeloid cells, enhanced differentiation of tumor-associated macrophages, and aberrant vascular hyperplasia (31–33). On the other hand, the high expression of PTPRZ1, BCAN, SEC61G, EGFR, and IGFBP2 in GSTCs, together with their overall higher stemness scores and transcriptional activity, collectively supports their characterization as the core malignant cell population. Previous studies have shown that EGFR signaling is involved not only in the proliferation and survival of central nervous system cells, but also in gliosis and regulation of the local tissue environment; in glioma, EGFR, IGFBP2, and SEC61G have also been associated with stem-like growth, immune evasion, and immune clearance escape in EGFR-amplified tumors, respectively (34–37). Therefore, the molecular features exhibited by GSTCs in this study are more likely to reflect not only the intrinsic growth advantages of tumor cells but also their capacity to adapt to, and potentially interact with, the surrounding microenvironment. Notably, we further observed that GSTCs were relatively more abundant in IDH-WT samples, whereas OSTCs were relatively more abundant in IDH-mut samples. This trend is consistent with previous reports showing reduced suppressive myeloid cells in IDH-mut gliomas, whereas IDH-WT gliomas are more invasive and accompanied by a more strongly immunosuppressive microenvironment (38, 39), suggesting that under the IDH-WT background, malignant cell states may be more tightly coupled to an inflammatory microenvironment accompanied by vascular remodeling.

Following further stratification within GSTCs, C0 VIM+ GSTCs and C3 CENPF+ GSTCs emerged as two biologically distinct states, thereby enabling a clearer distinction between microenvironment-adaptive programs and proliferation-dominant programs. The C0 subgroup was enriched for CHI3L1, VIM, LGALS3, VEGFA, and SEC61G. Previous studies have suggested that CHI3L1 is associated with tumor invasion and metastasis, high VIM expression is associated with glioma progression and immune infiltration, LGALS3 can promote the transition of glioblastoma toward a mesenchymal-like state, VEGFA is involved in angiogenesis and tumor growth, and SEC61G may contribute to immune evasion and tumor growth in GBM (37, 40–43). Together, these features support the interpretation that C0 VIM+ GSTCs represent a state characterized not only by invasiveness and mesenchymal-like features, but also by an increased propensity to engage with vascular–immune microenvironmental cues. In contrast, C3 CENPF+ GSTCs were mainly enriched for cell cycle- and mitosis-related genes such as TOP2A, CENPF, and NUSAP1 and thus more closely resembled a state dominated by proliferative advantage. Therefore, although both C0 and C3 were relatively increased in IDH-WT and both exhibited high stemness-related features, they represented distinct malignant programs: C3 was more biased toward a proliferative undifferentiated state, whereas C0 was more biased toward invasion, environmental response, and microenvironmental adaptation in a manner more relevant to vascular–immune crosstalk. Based on this distinction, C0 provides a more informative entry point for understanding IDH-WT-associated malignant progression and its coupling to the tumor microenvironment.

Functional profiling further reinforced this interpretation and more clearly positioned C0 within a vascular–immune-relevant malignant state framework. Compared with the other GSTC subgroups, C0 VIM+ GSTCs were overall more active in pathways such as angiogenesis, epithelial-mesenchymal transition, IL6/JAK/STAT3 signaling, and hypoxia, indicating that this subgroup is not merely a transcriptional subgroup defined by high VIM expression alone, but more likely represents a composite state formed by the superposition of multiple malignant programs. Previous studies have shown that, compared with IDH-mut gliomas, IDH-WT gliomas are typically accompanied by higher immune cell infiltration and angiogenesis; IL1B is associated with EMT-related phenotypic changes; and immune-high glioma subtypes generally exhibit higher EMT scores, whereas IDH mutation tends to be associated with lower immune levels (30, 44, 45). Meanwhile, IL6/JAK/STAT3 signaling and hypoxia can both promote tumor cell proliferation, survival, invasion, immunosuppression, and mesenchymal-like transformation, while collagen-enriched extracellular matrix can restrict immune cell infiltration through both physical and biochemical mechanisms and is also accompanied by enhanced angiogenic activity (46–50). Considering the molecular features and pathway activities corresponding to C0 in the present study, C0 is more appropriately understood as a malignant state that integrates inflammatory signaling responsiveness, hypoxic adaptation, mesenchymal-like transformation, and a tendency toward vascular remodeling, rather than simply reflecting enhanced proliferation alone. From a translational perspective, this state heterogeneity, together with its coupling to an immunosuppressive and angiogenic microenvironment, is also consistent with the current limitations of adoptive cell therapy in GBM, namely that substantial antigen heterogeneity and an immunosuppressive niche within solid tumors remain major barriers to therapeutic efficacy (51).

Compared with the composite malignant features of C0, the functional orientations of the other GSTC subgroups were relatively more focused. C3 CENPF+ GSTCs were mainly associated with processes such as cell cycle progression and active mitosis, which is consistent with the pathological characteristic of high mitotic activity in glioma (52). In contrast, C4 DCX+ GSTCs were enriched for features such as neuron, axonogenesis, projection, and differentiation and were therefore more consistent with a state characterized by neural development-like programs and neural lineage differentiation tendency. This interpretation is also in line with observations in GBM showing that ANAPC11 downregulation is accompanied by upregulation of neuron-related genes, cell cycle exit, and neuron-like differentiation (53–55). In comparison with these subgroups, which can be more readily defined in terms of “proliferation” or “differentiation,” the core changes in C0 were more concentrated in processes such as migration, assembly, stress, and localization. Its GO-BP enrichment results showed that C0 was mainly associated with oxidative phosphorylation, aerobic respiration, ATP synthesis coupled electron transport, and cellular respiration, suggesting that this subgroup may be accompanied by more active mitochondrial respiration and energy metabolism (56). At the same time, terms at the GO-MF and GO-CC levels, including cadherin binding, GTPase activity, focal adhesion, and cell-substrate junction, further support the notion that this subgroup exhibits more pronounced features of cell adhesion regulation, cytoskeletal dynamics remodeling, and enhanced cell-matrix interaction, all of which are closely related to glioma cell polarity changes, migratory invasion, and microenvironmental adaptation (1, 57, 58). Therefore, C0 more closely resembles a GSTC state with invasive bias, mesenchymal-like features, and a strong capacity to respond to microenvironmental cues.

From the perspective of state dynamics, GSTCs are better understood as a continuum of changing states rather than as mutually discrete fixed subgroups, which is particularly important when interpreting state-dependent interactions with the surrounding microenvironment. CytoTRACE showed that C3 CENPF+ GSTCs had the highest undifferentiated potential, whereas C0 VIM+ GSTCs also maintained a relatively high level, suggesting that both occupy a highly plastic range; however, the former is more consistent with a proliferative undifferentiated state supported by active cell cycle programs, whereas the latter combines a certain degree of plasticity with more evident environmental response features. Monocle and Slingshot further suggested that continuous state transitions with branching tendencies exist within GSTCs, in which C0 is more often located in the early-to-middle segment and connects to subsequent divergent trajectories, whereas C1 DLL3+ GSTCs and C2 XIST+ GSTCs are more biased toward the later part of the trajectory. It should be emphasized that CytoTRACE is more focused on evaluating the relative degree of undifferentiation, whereas pseudotime analysis places greater emphasis on the relative transition relationships among cell states; therefore, the two approaches do not address the same question. Taken together, these results more strongly support interpreting C0 as a highly plastic subgroup positioned at a key transition node within GSTCs, from which microenvironment-responsive and potentially vascular–immune-relevant malignant programs may emerge, rather than as an “early” cell population defined simply by proliferative advantage.

Glioma progression is highly dependent on sustained interactions between malignant cells and the tumor microenvironment, and myeloid cells are among the most important microenvironmental components in this context (59, 60). In recent years, studies of intercellular interactions within the tumor microenvironment have continued to focus on components such as macrophages, fibroblasts, and mesenchymal stem cells; for example, the rapid growth of research on exosomal circRNAs reflects the importance of this direction (61). In light of the relatively prominent communication links between C0 VIM+ GSTCs and myeloid cells observed in the present study, together with the tendency of C0 to act more as a receiver within the overall network, myeloid-derived IL6 family-related signals appear particularly relevant for linking inflammatory input to this IDH-WT-enriched malignant state. LIF/LIFR signaling has been shown to be associated with tumor growth, progression, metastasis, stemness, and treatment resistance, while IL6ST/gp130, as the common signal-transducing receptor chain of the IL6 family, represents a major hub in this class of pathways (62, 63). Previous studies have also suggested that both IL6 and IL11 are associated with tumor progression, angiogenesis, and immune evasion; in non-neoplastic central inflammatory models, alterations in astrocyte-derived IL11 can also affect microglial activation and migration through IL11Rα/gp130-related signaling (64–66). Therefore, in the present study, given the observed receptor basis of LIFR/IL6ST on the C0 side together with the OSM input directed toward it from myeloid cells, it is most appropriate to interpret this finding within a broader framework of IL6 family-mediated inflammatory input operating within a vascular–immune microenvironmental context; however, the current evidence is not sufficient to extrapolate IL11 itself as a direct participant in the cohort studied here, and this distinction still requires caution.

Within this context, OSM-related signaling is particularly noteworthy because it provides a plausible route through which myeloid-derived inflammatory cues may be coupled to the mesenchymal-like and angiogenesis-associated features of C0. Previous studies have shown that macrophage/myeloid-derived OSM can activate STAT3 through gp130-related receptor complexes containing OSMR or LIFR and induce a mesenchymal-like state in glioblastoma; OSM-mediated signaling can also promote malignant phenotypes associated with mesenchymal-like features through STAT3, enhance migratory and invasive capacity, and promote EMT-related invasion, metastasis, and the maintenance of cancer stem cell populations via the JAK/STAT3 and PI3K/AKT/mTOR pathways (67–69). Therefore, together with the myeloid-to-C0 OSM input predicted by CellChat analysis in the present study and the detectable LIFR/IL6ST receptor basis on the C0 side, we propose that myeloid-derived OSM signaling may represent a candidate pathway through which inflammatory microenvironmental input is linked to the mesenchymal-like, invasion-related, and vascular–immune-relevant phenotype of C0. Importantly, this interpretation should be regarded as a computationally inferred hypothesis based on expression patterns and network structure. It does not establish direct ligand transfer, spatial adjacency, receptor activation, or a causal OSM–STAT3–CEBPD signaling cascade *in situ*. This inference provides a direction for subsequent validation centered on STAT3 and downstream state regulation, but functional co-culture experiments, OSM perturbation, receptor blockade, and spatially resolved validation will be required to determine whether this predicted communication axis is mechanistically active. As a broader translational implication, cross-tumor studies have also suggested that the functional plasticity of immune effector cells itself may serve as an interventional entry point; for example, exogenous physical stimulation can enhance Wnt/β-catenin activity in NK cells and increase granzyme/perforin secretion (70). However, the applicability of such strategies in glioma still requires separate validation.

At the level of endogenous regulation, pySCENIC analysis suggested that C0 is not driven by a single regulator, but instead corresponds to an organized regulon landscape consistent with a coordinated state program rather than an isolated transcriptional event. RUNX2, POU2F3, TEAD3, and TEAD4 all have clear biological significance and are associated with EMT/malignant progression, lineage dependency, and processes related to metabolism or therapeutic resistance, respectively (71–75). This indicates that the regulatory network underlying C0 is multidimensional rather than changing along a single axis. Among these candidate factors, CEBPD merits priority attention not because the other factors are unimportant, but because it can be integrated more directly into the central framework of this study, namely the coupling of invasiveness, environmental adaptation, and inflammation-related microenvironmental input. CEBPD itself is highly context-dependent, acting as either an oncogenic factor or a tumor-suppressive factor in different cancer types and microenvironments, while also participating in inflammatory regulation, neuroinflammation, and certain central nervous system diseases (76). This context dependence fits well with the analytical framework of the present study, because we did not treat all gliomas as a homogeneous disease, but instead focused, within the comparative framework of IDH-WT and IDH-mut, on the C0 state that is more biased toward IDH-WT. Previous studies have shown that, in the hypoxic response of glioblastoma, CEBPD can promote EGFR phosphorylation and downstream PI3K pathway activation through fibronectin-mediated ECM-integrin signaling, thereby enhancing tumor cell invasiveness (77). This evidence is highly consistent, in direction, with the hypoxic adaptation, mesenchymal-like transformation, and enhanced cell-matrix interaction exhibited by C0 in the present study, making CEBPD more appropriately understood as a state-dependent regulatory node potentially linking microenvironment-associated signaling to invasive output and tissue remodeling.

If CEBPD is further considered within the framework of inflammatory factors and STAT3-related signaling, its logical connection to the above-mentioned myeloid input becomes clearer. Previous studies have shown that, in urothelial carcinoma of the bladder, cisplatin can induce CEBPD expression through the EGFR/STAT3 axis, thereby promoting the upregulation of resistance-related molecules; in low-grade glioma, high TMEM71 expression is associated with JAK2/STAT3 pathway activation and CEBPD upregulation; in addition, IL6 can promote STAT3 phosphorylation and upregulate Cebpd expression in bone-related cells through both cis- and trans-signaling (78–80). Although these lines of evidence are derived from different tumor types or tissue contexts, they consistently support the notion that CEBPD may occupy a connecting position between inflammatory factor input, growth factor receptor signaling, and downstream malignant phenotypes. In the context of the present study, the importance of this point lies in the fact that we are not merely observing that a given transcription factor is “upregulated,” but are instead attempting to explain why a C0 state biased toward IDH-WT simultaneously exhibits inflammatory signaling responsiveness, hypoxic adaptation, enhanced invasiveness, and close coupling to a vascular–immune microenvironmental context. Along this line of reasoning, CEBPD can be incorporated more readily than the other candidate regulatory factors into a unified mechanistic framework connecting myeloid input, state regulation, and malignant phenotypic output.

The *in vitro* functional experiments further suggest that CEBPD is phenotypically relevant in glioma cell-line models. Upon CEBPD downregulation, the clonogenic capacity, proliferative activity, and migratory ability of LN229 and U251 cells were reduced, while apoptosis was increased. These findings support the involvement of CEBPD in malignant phenotypic output in conventional glioma cells and are directionally consistent with the single-cell regulon analysis that nominated CEBPD as a candidate regulatory node associated with the C0 VIM+ GSTC state. However, these experiments should not be interpreted as direct validation that LN229 and U251 faithfully reproduce the C0 state, nor do they establish CEBPD as a fully validated master regulator of this patient-derived GSTC population. In conjunction with the single-cell analytical results, CEBPD is therefore more appropriately understood as a candidate effector that may connect C0-related transcriptional features with proliferative, survival, and invasive behavior, pending further validation in patient-derived GSTC models or organoid systems. From a broader survival signaling perspective, the PI3K/AKT/mTOR axis is widely involved in the regulation of tumor cell growth, proliferation, apoptosis, and metabolism, and its inhibition is often accompanied by restricted proliferation and enhanced apoptosis (81, 82). Therefore, the reduced colony formation, attenuated proliferation, and increased apoptosis observed after CEBPD downregulation in this study also suggest that future work could examine whether these effects are accompanied by changes in the activity of this pathway; however, at the current stage, we cannot directly attribute the effects of CEBPD to PI3K/AKT/mTOR inactivation on this basis alone.

Finally, several boundaries of the present study should be acknowledged. First, the discussion regarding the relationship among myeloid OSM input, STAT3-related signaling, and CEBPD is based mainly on computational inference from publicly available single-cell data and CEBPD knockdown experiments in conventional glioma cell lines. Although these results are mutually supportive at the level of biological direction, they still lack spatially resolved *in situ* evidence and functional validation of direct interactions between myeloid cells and C0-like tumor cells. Therefore, the OSM-STAT3-CEBPD framework proposed here should be regarded as a directional mechanistic hypothesis rather than a causal pathway that has already been directly demonstrated. Second, LN229 and U251 are useful and commonly used glioma cell-line models for preliminary perturbation experiments, but they cannot fully represent the transcriptomic and phenotypic complexity of the computationally defined C0 VIM+ GSTC state. In particular, the present study did not perform systematic molecular matching of these cell lines to the C0 signature, including IDH status and the expression levels of VIM, CHI3L1, VEGFA, and other state-associated markers. Future studies using patient-derived glioma stem-like cells, IDH-stratified models, or glioma organoids will be required to validate whether CEBPD plays a comparable role in bona fide C0-like tumor-cell states. Third, the mitochondrial gene filtering threshold of 25% was selected to avoid excessive exclusion of metabolically active or stress-adapted tumor cells from glioma tissues; nevertheless, we cannot fully exclude the possibility that some stress-associated cells were retained. Future sensitivity analyses using more stringent mitochondrial thresholds will help determine the robustness of the hypoxia- and angiogenesis-related findings. Fourth, the present single-cell analysis was mainly based on GSE278456. This dataset contains glioma samples across different IDH molecular backgrounds and allows malignant-cell states and microenvironmental components to be analyzed within a unified processing framework, thereby reducing additional batch effects that may arise from cross-platform integration. However, reliance on a single dataset limits the ability to establish broad generalizability. Independent validation in additional public glioma single-cell cohorts and patient-derived datasets will be necessary to confirm the reproducibility of the C0 VIM+ GSTC state and its association with CEBPD.

Overall, the present study supports the view that GSTCs are not a homogeneous population across different IDH backgrounds, and that one relatively IDH-WT-enriched VIM+ state may integrate mesenchymal-like transformation, hypoxic adaptation, angiogenesis, and inflammation-related microenvironmental input, thereby linking this state to a vascular–immune microenvironmental context, while CEBPD represents a candidate regulatory node with experimentally supported phenotypic relevance in glioma cell-line models.

## Data Availability

The single-cell RNA-sequencing data analyzed in this study are publicly available in the Gene Expression Omnibus (GEO) repository under accession number GSE278456. The remaining original contributions presented in the study are included in the article and Supplementary Material. Further inquiries can be directed to the corresponding authors.

## Supplementary material

Supplementary Figure 1 Initial clustering results and inferCNV-assisted identification of GSTCs and OSTCs. **(A)** Initial UMAP clustering of all cells, identifying a total of 35 Seurat clusters. **(B–E)** inferCNV heatmaps showing the copy number variation (CNV) patterns of cell clusters corresponding to GSTCs. Using cluster 26, corresponding to endothelial cells, as the reference population, the CNV features of cluster 1/3 **(B)**, cluster 5/6 **(C)**, cluster 10/7 **(D)**, and cluster 15/21/22/33/34/35 **(E)** are shown, respectively. **(F)** inferCNV heatmap showing the CNV patterns of cell clusters corresponding to OSTCs, including the copy number alteration features of cluster 11/27/29/30/32/8, with cluster 26 again used as the reference population. In the heatmaps, red indicates relative copy number gain and blue indicates relative copy number loss. Compared with the reference population, the cell clusters corresponding to GSTCs and OSTCs both exhibited varying degrees of large-scale chromosomal copy number alterations. These patterns helped identify cell populations with malignant features and provided supportive evidence for the subsequent classification of GSTCs and OSTCs.

Supplementary Figure 2 qRT-PCR validation of CEBPD knockdown efficiency in glioma cells. **(A)** Relative CEBPD mRNA expression in U251 and LN229 cells transfected with si-Ctrl, si-CEBPD#1, or si-CEBPD#2. Compared with the control group, both siRNAs markedly reduced CEBPD expression in the two cell lines. Data are presented as mean ± SD from three independent experiments. ***P* < 0.01.

Supplementary Table 1 Sample characteristics of the GSE278456 dataset, including sample ID, IDH status, WHO tumor grade, pathological diagnosis, cell numbers before and after quality control, and available clinical information.

Supplementary Table 2 Sequences of the CEBPD-targeting siRNAs and qRT-PCR primers used in this study. si-Ctrl represents the vendor-provided non-targeting control siRNA.
