# Supplementary material for: Single-cell analysis identifies a VIM+ glioma stem-like vascular–immune state linked to myeloid OSM signaling and CEBPD activity
Source: Front Immunol. 2026 Aug 11;17:1863737. doi: 10.3389/fimmu.2026.1863737 (PMC13503575; doi:10.3389/fimmu.2026.1863737)
Supplement: Supplementary file 1 [file Table1.docx]

**Supplementary Table 1. Sample characteristics of the GSE278456 dataset.**

| Sample Number | Patient Number | Tissue | Age | Sex | IDH | Diagnosis | Grade of tumor | Grade | Before quality control | After quality control |
| --- | --- | --- | --- | --- | --- | --- | --- | --- | --- | --- |
| GSM8546887_GBM036_Myl | GSM8546887_GBM036 | Brain | 60 | Female | IDH_WT | Glioblastoma | IV | High | 11456 | 9964 |
| GSM8546887_GBM036_Tumor | GSM8546887_GBM036 | Brain | 60 | Female | IDH_WT | Glioblastoma | IV | High |  |  |
| GSM8546896_GBM052_Myl | GSM8546896_GBM052 | Brain | 39 | Male | IDH_WT | Glioblastoma | IV | High | 17963 | 15096 |
| GSM8546896_GBM052_Tumor | GSM8546896_GBM052 | Brain | 39 | Male | IDH_WT | Glioblastoma | IV | High |  |  |
| GSM8546897_GBM054_Myl | GSM8546897_GBM054 | Brain | 63 | Female | IDH_WT | Glioblastoma | IV | High | 22111 | 17074 |
| GSM8546897_GBM054_Tumor | GSM8546897_GBM054 | Brain | 63 | Female | IDH_WT | Glioblastoma | IV | High |  |  |
| GSM8546898_GBM055_Myl | GSM8546898_GBM055 | Brain | 36 | Male | IDH_mut | Astrocytoma | III | High | 18389 | 13797 |
| GSM8546898_GBM055_Tumor | GSM8546898_GBM055 | Brain | 36 | Male | IDH_mut | Astrocytoma | III | High |  |  |
| GSM8546899_GBM056_Myl | GSM8546899_GBM056 | Brain | 77 | Female | IDH_WT | Glioblastoma | IV | High | 21463 | 16251 |
| GSM8546899_GBM056_Tumor | GSM8546899_GBM056 | Brain | 77 | Female | IDH_WT | Glioblastoma | IV | High |  |  |
| GSM8546901_GBM059_Myl | GSM8546901_GBM059 | Brain | 37 | Female | IDH_mut | Oligo | II | Low | 16551 | 12800 |
| GSM8546901_GBM059_Tumor | GSM8546901_GBM059 | Brain | 37 | Female | IDH_mut | Oligo | II | Low |  |  |
| GSM8546902_GBM060_Myl | GSM8546902_GBM060 | Brain | 30 | Female | IDH_mut | Astrocytoma | III | High | 24201 | 20218 |
| GSM8546902_GBM060_Tumor | GSM8546902_GBM060 | Brain | 30 | Female | IDH_mut | Astrocytoma | III | High |  |  |
| GSM8546905_GBM066_Myl | GSM8546905_GBM066 | Brain | 54 | Male | IDH_WT | Glioblastoma | IV | High | 13980 | 11514 |
| GSM8546905_GBM066_Tumor | GSM8546905_GBM066 | Brain | 54 | Male | IDH_WT | Glioblastoma | IV | High |  |  |
| GSM8546906_GBM068_Myl | GSM8546906_GBM068 | Brain | 42 | Female | IDH_mut | Oligo | II | Low | 24887 | 21230 |
| GSM8546906_GBM068_Tumor | GSM8546906_GBM068 | Brain | 42 | Female | IDH_mut | Oligo | II | Low |  |  |
| GSM8546910_GBM074_Myl | GSM8546910_GBM074 | Brain | 63 | Female | IDH_mut | Oligo | II | Low | 19361 | 16763 |
| GSM8546910_GBM074_Tumor | GSM8546910_GBM074 | Brain | 63 | Female | IDH_mut | Oligo | II | Low |  |  |
| GSM8546911_GBM075_Myl | GSM8546911_GBM075 | Brain | 30 | Female | IDH_mut | Astrocytoma | III | High | 19692 | 11237 |
| GSM8546911_GBM075_Tumor | GSM8546911_GBM075 | Brain | 30 | Female | IDH_mut | Astrocytoma | III | High |  |  |
| GSM8546915_GBM082_Myl | GSM8546915_GBM082 | Brain | 83 | Female | IDH_WT | Glioblastoma | IV | High | 24164 | 17175 |
| GSM8546915_GBM082_Tumor2 | GSM8546915_GBM082 | Brain | 83 | Female | IDH_WT | Glioblastoma | IV | High |  |  |
| GSM8546915_GBM082_Tumor | GSM8546915_GBM082 | Brain | 83 | Female | IDH_WT | Glioblastoma | IV | High |  |  |

Note: The last two columns show cell numbers before and after quality control.
