# Supplementary material for: Single-cell analysis identifies a VIM+ glioma stem-like vascular–immune state linked to myeloid OSM signaling and CEBPD activity
Source: Front Immunol. 2026 Aug 11;17:1863737. doi: 10.3389/fimmu.2026.1863737 (PMC13503575; doi:10.3389/fimmu.2026.1863737)
Supplement: Supplementary file 2 [file Table2.docx]

**Supplementary Table 2. Sequences of the siRNAs and qRT-PCR primers used in this study.**

| **Category** | **Name** | **Sequence (5′→3′)** |
| --- | --- | --- |
| siRNA | si-CEBPD#1 sense | UUCUCUCGCAGUUUAGUGGUG |
| siRNA | si-CEBPD#1 antisense | CACCACUAAACUGCGAGAGAA |
| siRNA | si-CEBPD#2 sense | AUUGCUGUUGAAGAGGUCGGC |
| siRNA | si-CEBPD#2 antisense | GCCGACCUCUUCAACAGCAAU |
| siRNA | si-Ctrl | vendor-provided non-targeting control |
| qRT–PCR primer | CEBPD-F (set A) | CCCCGCCATGTACGACGAC |
| qRT–PCR primer | CEBPD-R (set A) | CCCGCCTTGTGATTGCTGT |
| qRT–PCR primer | CEBPD-F (set B) | TCCGGCAGTTCTTCAAGCAGCT |
| qRT–PCR primer | CEBPD-R (set B) | GAGGTATGGGTCGTTGCTGAGT |
| qRT–PCR primer | ACTB-F | GGCACCCAGCACAATGAA |
| qRT–PCR primer | ACTB-R | TAGAAGCATTTGCGGTGG |
